# Hematopoietic Stem and Progenitor Cell Maintenance and Multiple Lineage Differentiation Is an Integral Function of NFATc1

**DOI:** 10.3390/cells11132012

**Published:** 2022-06-23

**Authors:** Carlotta Barahona de Brito, Stefan Klein-Hessling, Edgar Serfling, Amiya Kumar Patra

**Affiliations:** 1 Peninsula Medical School, University of Plymouth, Plymouth PL6 8BU, UK; carlotta.barahonadebrito@plymouth.ac.uk; 2 Department of Biology and Biochemistry, University of Bath, Bath BA2 7AX, UK; 3 Department of Molecular Pathology, Institute of Pathology, University of Wuerzburg, Josef Schneider-Str. 2, 97080 Wuerzburg, Germany; stefan.klein-hessling@uni-wuerzburg.de (S.K.-H.); serfling.e@uni-wuerzburg.de (E.S.); 4 Comprehensive Cancer Center Mainfranken, University of Wuerzburg, Josef Schneider-Str. 6, 97080 Wuerzburg, Germany

**Keywords:** hematopoiesis, HSC, lineage differentiation, NFATc1

## Abstract

Hematopoietic stem and progenitor cell (HSPC) maintenance and the differentiation of various lineages is a highly complex but precisely regulated process. Multiple signaling pathways and an array of transcription factors influence HSPC maintenance and the differentiation of individual lineages to constitute a functional hematopoietic system. Nuclear factor of activated T cell (NFAT) family transcription factors have been studied in the context of development and function of multiple mature hematopoietic lineage cells. However, until now their contribution in HSPC physiology and HSPC differentiation to multiple hematopoietic lineages has remained poorly understood. Here, we show that NFAT proteins, specifically NFATc1, play an indispensable role in the maintenance of HSPCs. In the absence of NFATc1, very few HSPCs develop in the bone marrow, which are functionally defective. In addition to HSPC maintenance, NFATc1 also critically regulates differentiation of lymphoid, myeloid, and erythroid lineage cells from HSPCs. Deficiency of NFATc1 strongly impaired, while enhanced NFATc1 activity augmented, the differentiation of these lineages, which further attested to the vital involvement of NFATc1 in regulating hematopoiesis. Hematopoietic defects due to lack of NFATc1 activity can lead to severe pathologies such as lymphopenia, myelopenia, and a drastically reduced lifespan underlining the critical role NFATc1 plays in HSPC maintenance and in the differentaion of various lineages. Our findings suggest that NFATc1 is a critical component of the myriad signaling and transcriptional regulators that are essential to maintain normal hematopoiesis.

## 1. Introduction

Hematopoiesis is a complex but precisely regulated process through which hematopoietic stem and progenitor cells (HSPC) give rise to mature blood cells of all lineages [1,2]. Hematopietic stem cells (HSCs) remain at the top of the hierarchy, which have the unique ability to self-renew in a controlled manner and also, to differentiate to the immediate downstream progenitor cells of specific lineages. Because of the regulated proliferation, HSCs are mostly quiescent, which is a characteristic feature of the stemness of these cells. In the bone marrow (BM), HSCs reside in a very specialized environment, called the hematopoietic stem cell niche [3,4,5]. There, an array of intrinsic and extrinsic signaling pathways and multiple transcription factors (TFs) regulate HSPC maintenance [6,7,8]. HSPCs are identified as Lin^−^Sca1^+^c-Kit^+^ (LSK) cells, which are lineage marker negative and express both the stem cell antigen-1 (Sca1) and stem cell factor receptor (c-Kit/CD117). Further evaluation of LSK cells based on the expression of the signaling lymphocyte activation molecule (SLAM) family receptors defines HSCs as LSKCD150^+^ cells [9]. Based on their functional properties, HSCs consist of two distinct populations. LSK cells lack the expression of fetal liver kinase-2 (Flk-2) and are the long-term repopulating stem cells (LT-HSCs), which are mostly quiescent and undergo limited cell cycle for self-renewal [10]. LSK cells have high expression of Flk-2 and are the short-term repopulating stem cells (ST-HSCs), which have limited ability to self-renew but they differentiate to give rise to the common lymphoid progenitor (CLP), common myeloid progenitor (CMP), and the megakaryocyte-erythrocyte progenitor (MEP) populations. These progenitor populations occupy specific hematopoietic niches to give rise to mature blood cells of all lineages.

CLP-derived T cells develop in the thymus, whereas B cells and NK cells are produced in the BM. All myeloid cells such as dendritic cells (DCs), macrophages, monocytes, granulocytes, and neutrophils are derived from the CMPs in the BM. Whereas lymphocytes are the major components of the adaptive immune system, cells of the myeloid origin constitute the innate immune system. In addition, in the BM, MEPs differentiate to produce erythrocytes and megakaryocytes, which are vital for the survival of an organism. All these components that are responsible for specific functions, together, form the hematopoietic system. Although various TFs have been implicated to regulate HSPC differentiation and maintenance [6,11], the role of the nuclear factor of activated T cells (NFAT) family TFs in this process has not been explored yet. The NFAT family consists of five members, i.e., NFATc1, NFATc2, NFATc3, NFATc4, and NFAT5. Except for NFAT5, the other family members are activated in a calcium- and serine/threonine phosphatase calcineurin-dependent manner [12,13]. We recently reported a calcium and calcineurin-independent mechanism of NFAT activation, which played a critical role in T cell development [14]. Previous studies have shown the critical involvement of NFAT proteins in the development, differentiation, and function of various mature lineage-positive cells [15,16,17]. However, whether NFAT proteins together or any specific family member individually play any role in the maintenance of HSPCs, and whether they can influence hematopoiesis in addition to their roles in the already differentiated lineage-positive cells is an interesting aspect to be investigated.

Using a murine model where NFATc1 activity was specifically ablated in a hematopoietic system (*Vav*-Cre*Nfatc1*^fl/fl^ mice), we recently showed that it played an indispensable role in T and B cell development in the thymus and BM, respectively [14,18,19]. Mice deficient in the activity of other NFAT family members did not show a similar phenotype. In addition, in *Vav*-Cre*Nfatc1*^fl/fl^ mice, the presence of other NFAT proteins did not compensate for the loss of NFATc1 activity in lymphocyte development [14]. NFATc1-deficiency in these mice also impaired erythropoiesis [20], suggesting that NFATc1 is a key component of the regulatory mechanism that contributes towards normal development of various hematopoietic lineages. These observed strong influences of NFATc1 deficiency on multiple hematopoietic lineages led us to investigate whether it also regulates the maintenance of HSPCs. Here, we show that NFATc1 is the key family member that exerts vital control over HSPCs, and also on the differentiation of other lineage-positive cells, which, so far, has been unknown. We demonstrate that NFATc1 is expressed right from the HSCs down to all progenitor populations and in lineage-positive cells, and NFATc1 deficiency leads to severe abnormalities in HSPC maintenance, which strongly affects hematopoiesis.

## 2. Materials and Methods

### 2.1. Mice

C57BL/6 wild-type, Nfatc1^fl/fl^, Vav-CreNfatc1^+/fl^, Vav-CreNfatc1^fl/fl^, Nfatc2^−/−^, Nfatc3^−/−^, Nfatc2^−/−^Nfatc3^−/−^, Nfatc1-eGfp-Bac tg, Nfatc1P2^fl/fl^, Vav-CreNfatc1P2^fl/fl^, Nfatc1αA^fl/fl^, Vav-creNfatc1αA^fl/fl^, Il2^+/−^, Il2^−/−^, and Il2^−/−^Nfatc1-eGfp-Bac tg mice, on C57BL/6 background and 3–8 weeks of age unless mentioned otherwise were used throughout the study. Animals were housed in the central animal facility (ZEMM) of the University of Würzburg, according to standard animal care protocols. All animal experiments were performed taking utmost care, and were according to established guidelines (approved by the Regierung von Unterfranken, Wuerzburg, Germany permit number 55.2-2531.01-53/10B to E.S.).

### 2.2. Flow Cytometry

All antibodies used in flow cytometry, and in cell sorting were purchased either from BD Pharmingen or eBioscience. Anti-Ly-6A/E (Sca1; D7), anti-c-Kit (2B8), Biotin mouse lineage panel (#559971), anti-CD45R/B220 (RA3-6B2), anti-CD3ε (145-2C11), anti-CD4 (GK1.5), anti-CD8α (53-6.7), anti-CD11a (2D7), anti-CD11b (M1/70), anti-CD11c (N418), anti-CD18 (C71/16), anti-CD25 (PC61), anti-CD41 (MWReg30), anti-CD45.1 (A20), anti-CD45.2 (104), anti-CD49d (R1-2), anti-Ly-6G (Gr1; RB6-8C5), anti-Ter119 (TER-119), anti-CD62L (MEL-14), anti-CD71 (C2), anti-CD127 (A7R34), anti-CD135 (Flk2; A2F10), anti-CD150 (9D1), anti-IgM (R6-60.2), and anti-IgD (11-26c) antibodies, either directly conjugated with fluorochromes or with biotin, were used throughout this study. Biotinylated antibodies were revealed with secondary streptavidin-allophycocyanin or phycoerythrin-Cy5 (PE-Cy5) antibodies. The flow cytometry and data analysis were performed following standard procedure using the FACSCalibur (Franklin Lakes, NJ, USA) and FlowJo software (Franklin Lakes, NJ, USA).

### 2.3. Cell Isolation and Sorting

For HSPC (Lin^−^Sca1^+^c-Kit^+^), Sca1^+^ (Lin^−^Sca1^+^c-Kit^−^), c-Kit^+^ (Lin^−^Sca1^−^c-Kit^+^), or L^−^S^−^K^−^ (Lin^−^Sca1^−^c-Kit^−^) cell sorting, single cell suspension of BM cells from both fore limbs and hind limbs were first depleted of lineage-positive cells using the Biotin Mouse Lineage Panel antibodies. Lineage-negative (Lin^−^) BM cells were isolated using anti-Biotin microbeads (Miltenyi Biotec) in magnetic separation following the manufacturer’s protocol. Lin^−^ cells were incubated with anti-Sca1 and anti-c-Kit antibodies, and HSPC (LSK), Sca1^+^, c-Kit^+^, or L^−^S^−^K^−^ cells were sorted by using a FACSAria (BD Biosciences) flow cytometer. For sorting of LT-HSC (Lin^−^IL-7R^−^Sca1^+^c-Kit^+^Flk2^−^), ST-HSC (Lin^−^IL-7R^−^Sca1^+^c-Kit^+^Flk2^+^), CLP (Lin^−^IL-7R^+^Sca1^−^c-Kit^lo^), CMP (Lin^−^IL-7R^−^Sca1^−^c-Kit^+^), GMP (Lin^−^IL-7R^−^Sca1^−^c-Kit^+^ CD34^+^CD16/32^hi^), and MEP (Lin^−^IL-7R^−^Sca1^−^c-Kit^+^CD34^−^CD16/32^lo^) cells, appropriate additional antibodies were added to the Lin^−^ BM cells in addition to anti-Sca1 and anti-c-Kit antibodies. Gr1^+^, CD11b^+^, and Ter119^+^ cells were sorted from BM cells and, CD4^+^ T and B220^+^ B cells were sorted from splenocytes of WT or *Vav*-Cre*Nfatc1P2*^fl/fl^ mice.

### 2.4. Immunofluorescence Staining

Sorted HSPC (LSK), Sca1^+^ (Lin^−^Sca1^+^c-Kit^−^), c-Kit^+^ (Lin^−^Sca1^−^c-Kit^+^), L^−^S^−^K^−^ (Lin^−^Sca1^−^c-Kit^−^), LT-HSCs, or ST-HSCs from WT mice were immunostained with NFATc1 (Santa Cruz, sc14034) antibodies following a previously published protocol [21]. Nuclear NFATc1 was confirmed by co-staining the cells with DAPI. Image acquisition and analysis were done with a TCS SP2 Leica confocal microscope and software. Nuclear NFATc1 in each immunofluorescence experiment was estimated on individual cells in each focus using the Adobe Photoshop software. The nuclear area was demarcated for each cell and the mean relative fluorescence was determined for the channel used using the histogram option. This was done for a number of cells in each focus and multiple foci from each experiment were analyzed. The mean for each group in each experimental replicate were calculated. These mean values in each group from three experimental replicates were used for the figures and for statistical analysis.

### 2.5. Adoptive Cell Transfer

Splenocytes (5 × 10^6^) depleted of RBCs, from CD45.2^+^ *Nfatc1*^fl/fl^ or *Vav*-Cre*Nfatc1*^fl/fl^ donors, were transferred to lethally (9 Gy) irradiated CD45.1^+^ congenic WT recipients by retro-orbital injection into the venous sinus. Post-adoptive transfer, recipient mice were maintained with antibiotic-supplemented drinking water and were observed carefully. Hematopoietic reconstitution in recipient mice was analyzed at 11 weeks after transfer by gating on donor-derived cells.

### 2.6. Semiquantitative RT-PCR

Sorted HSPC, LT-HSC, ST-HSC, CLP, CMP, GMP, MEP, Gr1^+^, CD11b^+^, Ter119^+^, Sca1^+^, c-Kit^+^, and L^−^S^−^K^−^ cells from BM of WT, *Nfatc1*^fl/fl^, *Vav*-Cre*Nfatc1*^+/fl^, *Vav*-Cre*Nfatc1*^fl/fl^, *Nfatc1P2*^fl/fl^, *Vav*-Cre*Nfatc1P2*^fl/fl^, or *Il2*^−/−^ mice; CD4^+^ T and B220^+^ B cells from spleen of WT; thymocytes and splenocytes from *Vav*-Cre*Nfatc1P2*^fl/fl^; and Lin^−^ BM cells from *Nfatc1*^fl/fl^ or *Vav*-Cre*Nfatc1*^fl/fl^ mice were used to synthesize cDNA using a Miltenyi Biotec cDNA synthesis kit and protocol. Semiquantitative RT-PCR was performed to reveal the level of expression of indicated genes. Primer sequences are available in the Supplementary Materials online.

### 2.7. Photographs

Photographs of *Nfatc1*^fl/fl^, *Vav*-Cre*Nfatc1*^+/fl^, and *Vav*-Cre*Nfatc1*^fl/fl^ mice for body size, tooth eruption, and the bones from hind limbs were taken using a Nikon Coolpix 4500 digital camera, and the photographs were processed using the Adobe Photoshop software.

### 2.8. Statistics

**Figure 1:**
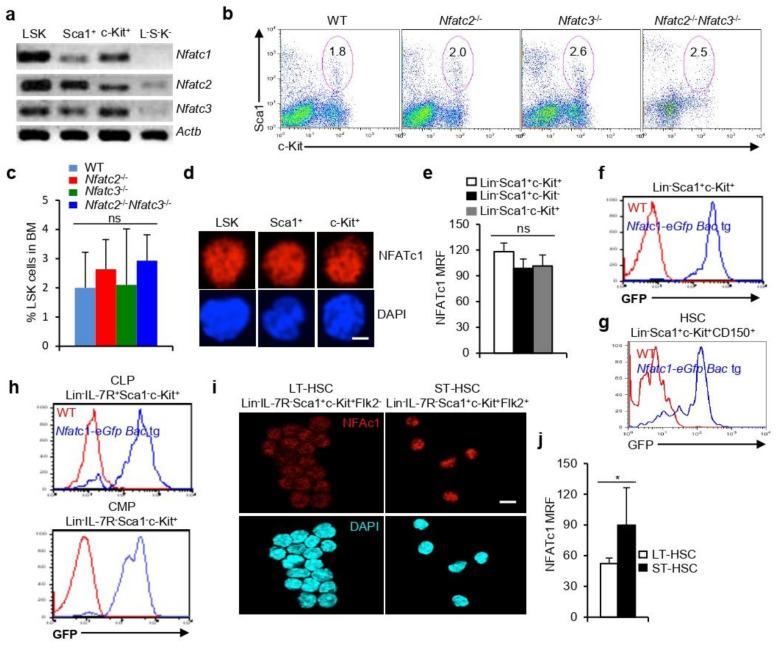
**NFAT expression in HSPCs**: (**a**) Expression of *Nfatc1*, *Nfatc2*, and *Nfatc3* in sorted BM Lin^−^Sca1^+^c-Kit^+^ (LSK), Lin^−^Sca1^+^c-Kit^−^ (Sca1^+^), Lin^−^Sca1^−^c-Kit^+^ (c-Kit^+^), and Lin^−^Sca1^−^c-Kit^−^ (L^−^S^−^K^−^) cells from WT mice; (**b**) flow cytometry profiles showing distribution of BM LSK cells in WT, *Nfatc2*^−/−^, *Nfatc3*^−/−^, and *Nfatc2*^−/−^*Nfatc3*^−/−^ mice. The number inside each dot plot represents percent LSK cells; (**c**) quantification of BM LSK cells distribution in indicated mice (WT, *n* = 11; *Nfatc2*^−/−^, *n* = 14; *Nfatc3*^−/−^, *n* = 3; and *Nfatc2*^−/−^*Nfatc3*^−/−^, *n* = 3); (**d**) immunofluorescence analysis for nuclear NFATc1 levels in sorted LSK, Sca1^+^, and c-Kit^+^ cells from WT mice. Co-staining with DAPI confirms nuclear NFATc1. Scale bar, 10 μm; (**e**) quantification of mean relative fluorescence (MRF) of nuclear NFATc1 levels in sorted BM LSK, Sca1^+^, and c-Kit^+^ cells from WT mice (LSK, *n* = 63; Sca1^+^, *n* = 73; and c-Kit^+^, *n* = 30 cells from three experimental replicates); (**f**) GFP expression representing NFATc1 levels in BM Lin^−^Sca1^+^c-Kit^+^ HSPCs from *Nfatc1-eGfp-Bac* tg reporter mice as compared with WT mice; (**g**) GFP expression representing NFATc1 levels in BM Lin^−^Sca1^+^c-Kit^+^CD150^+^ HSCs from *Nfatc1-eGfp-Bac* tg reporter mice as compared with WT mice; (**h**) GFP levels representing NFATc1 expression in BM Lin^−^IL-7R^+^Sca1^−^c-Kit^+^ CLP and Lin^−^IL-7R^−^Sca1^−^c-Kit^+^ CMP cells from *Nfatc1-eGfp-Bac* tg reporter mice as compared with WT mice; (**i**) immunofluorescence analysis for nuclear NFATc1 levels in sorted LT-HSC (Lin^−^Sca1^+^c-Kit^+^Flk2^−^) and ST-HSC (Lin^−^Sca1^+^c-Kit^+^Flk2^+^) from BM cells of WT mice. Co-staining with DAPI confirms nuclear NFATc1. Scale bar, 10 μm; (**j**) quantification of mean relative fluorescence (MRF) of nuclear NFATc1 levels in sorted BM LT- and ST-HSCs from WT mice. (LT-HSCs, *n* = 68 and ST-HSCs, *n* = 33 cells from three experimental replicates). Data are representative of three independent experiments, and in (**c**,**e**) (ns = not significant, one-way ANOVA) and in (**j**) (* *p* = 0.0209, unpaired *t*-test) are presented as mean ± s.d.

Data are presented as mean ± s.d. Statistical significance was assessed using Student’s *t*-test for comparisons between two groups and ANOVA for differences between groups. The *p*-values wherever applicable (Figure 1e,j) were calculated based on the means of the experimental replicates. The survival of *Vav*-Cre*Nfatc1*^fl/fl^ mice as compared with littermate control mice was analyzed by Kaplan–Meier survival plot.

## 3. Results

### 3.1. NFAT Expression in HSPC Population

To investigate whether NFAT family transcription factors play any roles in HSPC development and differentiation, we analyzed for *Nfat* expression in WT LSK cells (Supplementary Figure S1a). Expressions of *Nfatc1*, *Nfatc2*, and *Nfatc3*, the three major members of NFAT family, were readily detectable in HSPCs (Figure 1a). In addition to HSPCs, strong *Nfat* expression was also detectable in Lin^−^Sca1^−^c-Kit^+^ (c-Kit^+^) and Lin^−^Sca1^+^c-Kit^−^ (Sca1^+^) cells, suggesting that *Nfat* expression is not only present in HSPCs but also in all hematopoietic lineage cells (Figure 1a). A weak *Nfat* expression in Lin^−^Sca1^−^c-Kit^−^ (L^−^S^−^K^−^) cells further suggests that there is a differential expression of *Nfat* genes in various Lin^−^ BM cell populations (Figure 1a). To determine whether NFAT proteins combinatorially or individually regulate HSPC physiology, and to identify the key NFAT family member regulating HSPC function, we analyzed various *Nfat* gene ko mice. *Nfatc1* ko mice are embryonic lethal [22]. The analysis of mice deficient in NFATc2 (*Nfatc2*^−/−^), NFATc3 (*Nfatc3*^−/−^), or both (*Nfatc2*^−/−^*Nfatc3*^−/−^) revealed comparable proportion of HSPCs as compared with littermate control mice (Figure 1b,c). Further, the relatively normal phenotype of these ko mice suggests that NFATc1 is most likely the key family member that plays a role in HSPC biology.

In line with the strong *Nfatc1* expression, high levels of NFATc1 proteins were also detected in HSPC, c-Kit^+^, and Sca1^+^ cells (Figure 1d,e and Supplementary Figure S1b). The analysis of *Nfatc1-eGfp-Bac* tg reporter mice [23] further supported *Nfatc1* expression in HSPCs (Figure 1f), and also in HSCs (Figure 1g). Again, *Nfatc1* expression was not restricted to HSCs, but was also detected in the downstream common lymphoid progenitor (CLP, Lin^−^IL-7R^+^Sca1^−^c-Kit^+^) and common myeloid progenitor (CMP;,Lin^−^IL-7R^−^Sca1^−^c-Kit^+^) cells, suggesting that NFATc1 is widely expressed in the hematopoietic system (Figure 1h). Further, analysis of long- and short-term HSCs (Lin^−^Sca1^+^c-Kit^+^Flk2^−^ (LT-HSCs) and Lin^−^Sca1^+^c-Kit^+^Flk2^+^ (ST-HSCs)) revealed the presence of a higher level of NFATc1 proteins in ST-HSCs as compared with that in the LT-HSCs (Figure 1i,j).

### 3.2. NFATc1 Critically Regulates HSPC Development

**Figure 2:**
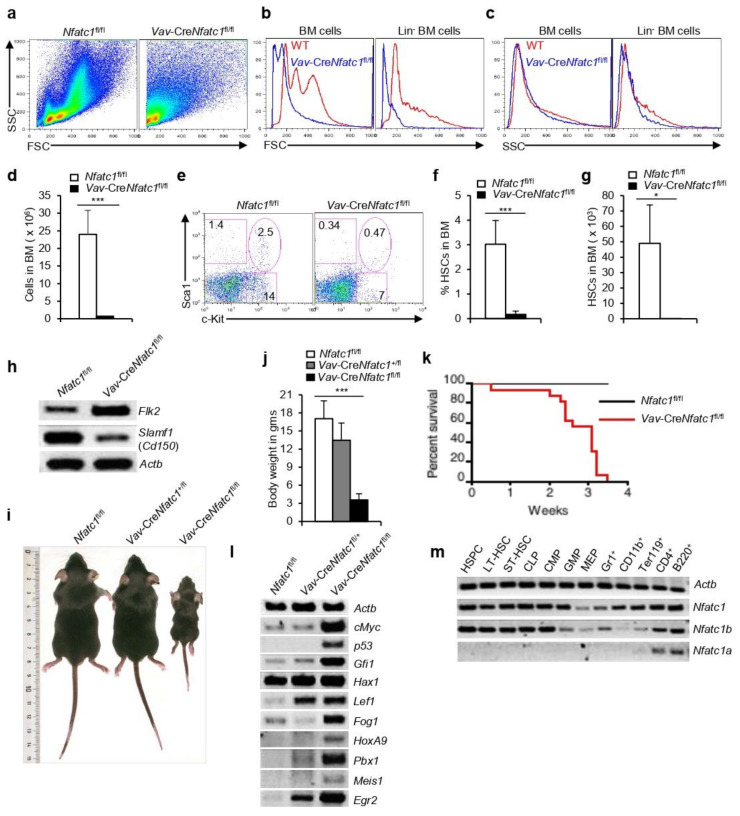
**Impaired HSPC development in *Vav*-Cre*Nfatc1*^fl/fl^ mice**: (**a**) Flow cytometry profiles of total BM cells based on FSC and SSC patterns from *Nfatc1*^fl/fl^ and *Vav*-Cre*Nfatc1*^fl/fl^ mice; (**b**) cell size based on FSC pattern of total and Lin^−^ BM cells from *Nfatc1*^fl/fl^ and *Vav*-Cre*Nfatc1*^fl/fl^ mice; (**c**) granularity in total and Lin^−^ BM cells based on SSC pattern from *Vav*-Cre*Nfatc1*^fl/fl^ mice as compared with *Nfatc1*^fl/fl^ controls; (**d**) total BM cells in *Vav*-Cre*Nfatc1*^fl/fl^ (*n* = 5) and *Nfatc1*^fl/fl^ (*n* = 10) mice; (**e**) flow cytometry profiles revealing LSK cells distribution in the BM of *Vav*-Cre*Nfatc1*^fl/fl^ mice as compared with littermate control mice. Total BM cells were used for the analysis and numbers inside each FACS plot represent percent respective populations; (**f**) quantification of percent BM LSK cells in *Vav*-Cre*Nfatc1*^fl/fl^ mice as compared with *Nfatc1*^fl/fl^ mice (*n* = 9 each); (**g**) quantification of BM LSK cells in *Vav*-Cre*Nfatc1*^fl/fl^ (*n* = 6) and *Nfatc1*^fl/fl^ (*n* = 7) mice; (**h**) levels of *Flk2* and *Slamf1* (*Cd150*) mRNA expression in sorted BM LSK cells from *Vav*-Cre*Nfatc1*^fl/fl^ and *Nfatc1*^fl/fl^ mice; (**i**) body size of *Vav*-Cre*Nfatc1*^fl/fl^ mice as compared with that of age-matched littermate control mice; (**j**) quantification of body weight of *Nfatc1*^fl/fl^ (*n* = 9), *Vav*-Cre*Nfatc1*^+/fl^ (*n* = 9), and *Vav*-Cre*Nfatc1*^fl/fl^ (*n* = 12) mice; (**k**) Kaplan–Meier survival curves for age-matched *Nfatc1*^fl/fl^ and *Vav*-Cre*Nfatc1*^fl/fl^ mice (*n* = 16 per group); (**l**) gene expression analysis of various TFs essential for HSC maintenance, quiescence, and proliferation in LSK cells from *Vav*-Cre*Nfatc1*^fl/fl^ mice as compared with littermate control mice; (**m**) RT-PCR analysis of the expression levels of total *Nfatc1*, and the *Nfatc1a* and *Nfatc1b* isoforms in sorted hematopoietic cell populations from WT mice BM. *Nfatc1*^fl/fl^, *Vav*-Cre*Nfatc1*^+/fl^, and *Vav*-Cre*Nfatc1*^fl/fl^ littermate mice were 3 weeks old and WT mice used in the experiments were 6 weeks old. Data are representative of three independent experiments, and in (**d**,**f**) (*** *p* < 0.0001, paired *t*-test), (**g**) (* *p* = 0.0215, unpaired *t*-test), and (**j**) (*** *p* < 0.0001, one-way ANOVA) are presented as mean ± s.d.

To unravel the role of NFATc1 in HSPC generation, we analyzed *Vav*-Cre*Nfatc1*^fl/fl^ mice, in which NFATc1 activity was ablated in the hematopoietic system [14]. The flow cytometry analysis of BM cells immediately revealed gross anomalies in the distribution of cells according to their FSC and SSC patterns. Whereas, in *Nfatc1*^fl/fl^ mice, both FSC^lo^SSC^lo^ and FSC^hi^SSC^hi^ cells were evident; in *Vav*-Cre*Nfatc1*^fl/fl^ mice, the BM cells were mostly FSC^lo^SSC^lo^, suggesting a defective hematopoiesis in absence of NFATc1 (Figure 2a). Due to the lack of NFATc1, *Vav*-Cre*Nfatc1*^fl/fl^ total BM cells as well as the Lin^−^ cells, which contain the HSPCs, were much smaller in size, although they were similar in their granularity as compared with the control cells (Figure 2b,c). Defective hematopoiesis in generating enough blood cells was evident as the BM cellularity in the absence of NFATc1 reduced drastically in *Vav*-Cre*Nfatc1*^fl/fl^ mice (Figure 2d). Accordingly, the analysis of the HSPC population in *Vav*-Cre*Nfatc1*^fl/fl^ mice revealed a strong decrease as compared with that in the littermate control mice (Figure 2e–g). Simultaneously, c-Kit^+^ and Sca1^+^ cells were also severely reduced in *Vav*-Cre*Nfatc1*^fl/fl^ mice (Figure 2e) indicating a severe defect in hematopoiesis in general in the absence of NFATc1 activity. In the BM, self-renewing LT-HSCs, which undergo limited cell cycle and are mostly quiescent, give rise to the ST-HSCs, which divide actively and differentiate to produce the progenitor populations of all hematopoietic lineages. Surprisingly, in *Vav*-Cre*Nfatc1*^fl/fl^ HSPCs, we observed a strong decrease in the expression of *Cd150* (*Slamf1*) and an increased expression of *Flk2* indicating an increased prevalence of ST-HSCs and a paucity of LT-HSCs as compared with littermate *Nfatc1*^fl/fl^ mice (Figure 2h). Hematopoietic loss of NFATc1 had a severe consequence on the life of *Vav*-Cre*Nfatc1*^fl/fl^ mice. These mice were not born with normal frequency (2.022% actual frequency against 12.5% expected). As compared with age- and gender-matched littermate control mice, the *Vav*-Cre*Nfatc1*^fl/fl^ mice were significantly smaller in their body size and weight (Figure 2i,j). This phenotype was already evident in *Vav*-Cre*Nfatc1*^fl/+^ mice (Figure 2j), suggesting that the effects of NFATc1 deficiency in the hematopoietic system, on the overall phenotype of these animals are profound. Interestingly, none of the *Vav*-Cre*Nfatc1*^fl/fl^ mice survived beyond 4 weeks after birth (Figure 2k). The number of HSPCs was reduced in *Vav*-Cre*Nfatc1*^fl/fl^ mice, and their gene expression patterns were also severely affected in the absence of NFATc1 activity. Gene expression of several of the key transcription factors and other molecules essential for HSC quiescence, survival, and function was not lacking, rather, it was strongly upregulated in *Vav*-Cre*Nfatc1*^fl/fl^ HSPCs (Figure 2l). The severe phenotype of *Vav*-Cre*Nfatc1*^fl/fl^ mice suggests a general failure of the hematopoietic system in the absence of NFATc1 activity, which has not been observed in the case of mice deficient in any other NFAT family members. In support of this hypothesis, *Nfatc1* expression was easily detected in HSPCs, various progenitor populations, as well as in mature hematopoietic cells (Figure 2m).

*Nfatc1* expression is directed from two distinct promoters, a distal P1 promoter directing the synthesis of the inducible NFATc1α isoforms, and a proximal P2 promoter initiating the synthesis of the constitutively expressed NFATc1β isoforms [24]. We recently reported the critical role of this promoter-specific *Nfatc1* expression during T and B cell differentiation in a developmental stage-dependent manner [18,19]. The analysis for any promoter-specific preferential *Nfatc1* expression in hematopoietic stem and progenitor cells, as well as in lineage-positive mature cell populations, revealed a distinct P2 promoter-induced *Nfatc1b* activity in all hematopoietic cells starting from the HSCs downwards (Figure 2m). P1-derived *Nfatc1a* isoform was detectable only in mature T and B cells, which express the TCR and BCR, respectively, along with *Nfatc1b* isoform (Figure 2m). We previously reported NFATc1 expression in lymphocytes and erythrocytes [14,18,19,20,25]. Analysis of various myeloid and erythroid populations from *Nfatc1-eGfp-Bac* tg mice further confirmed NFATc1 expression in these lineage-positive cells (Supplementary Figure S2), suggesting a pan-hematopoietic system-specific function of NFATc1.

### 3.3. HSPC-Specific Defects Result in Severe Lymphopenia in Vav-CreNfatc1^fl/fl^ Mice

**Figure 3:**
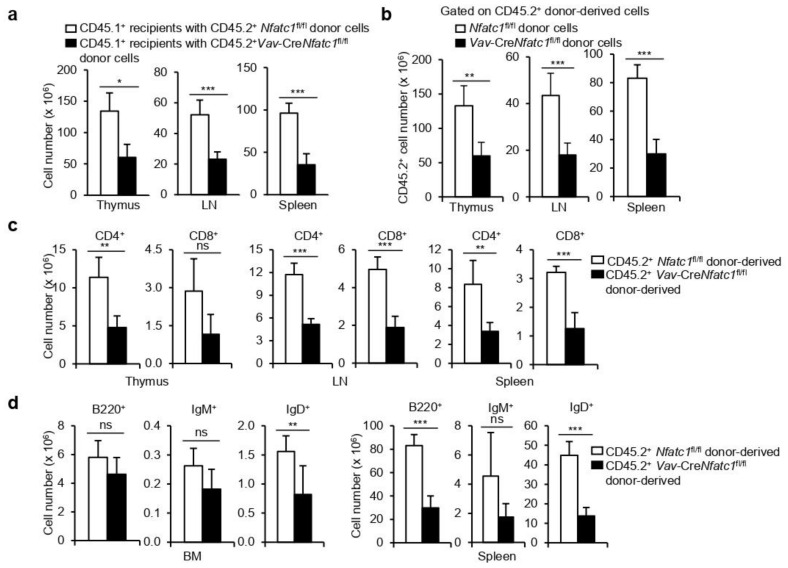
**Poor reconstitution capacity of NFATc1-deficient HSPCs**: (**a**) Cellularity in the thymus (* *p* = 0.0461), LNs (*** *p* = 0.0002), and spleen (*** *p* < 0.0001) of CD45.1^+^ recipient mice transferred with splenocytes from CD45.2^+^ *Nfatc1*^fl/fl^ or *Vav*-Cre*Nfatc1*^fl/fl^ mice; (**b**) CD45.2^+^ *Nfatc1*^fl/fl^ or *Vav*-Cre*Nfatc1*^fl/fl^ donor-derived cell numbers in the thymus (** *p* = 0.0059), LNs (*** *p* = 0.0005), and spleen (*** *p* < 0.0001) of CD45.1^+^ recipient mice; (**c**) number of CD45.2^+^ *Nfatc1*^fl/fl^ or *Vav*-Cre*Nfatc1*^fl/fl^ donor-derived CD4^+^ and CD8^+^ T cells in the thymus (** *p* = 0.0048, ns = not significant), LNs (*** *p* < 0.0001), and spleen (** *p* = 0.0021 and *** *p* = 0.0002) of CD45.1^+^ recipient mice; (**d**) Number of CD45.2^+^ *Nfatc1*^fl/fl^ or *Vav*-Cre*Nfatc1*^fl/fl^ donor-derived B220^+^, IgM^+^, and IgD^+^ B cells in the BM (ns = not significant and ** *p* = 0.0036) and spleen (ns = not significant and *** *p* < 0.0001) of CD45.1^+^ recipient mice. The number of recipient mice used in each group was three per experiment. Data are representative of two independent experiments and are presented as mean ± s.d., unpaired *t*-test.

We recently reported severely impaired T and B cell development in *Vav*-Cre*Nfatc1*^fl/fl^ mice due to defects in the thymus and BM, respectively, at the ealiest stages of differentiation. These defects invariably led to strong T and B cell lymphopenia in these mice (14, 18, 19). The development of similar pathological conditions in two distinct lymphoid lineage cells lacking NFATc1 activity led us to investigate whether additional defects at the HSPC level also contribute to these phenotypes in *Vav*-Cre*Nfatc1*^fl/fl^ mice. To delineate any HSPC-specific defects in T and B lymphopoiesis, we adoptively transferred CD45.2^+^ *Nfatc1*^fl/fl^ or *Vav*-Cre*Nfatc1*^fl/fl^ splenocytes to lethally irradiated congenic CD45.1^+^ hosts. Eleven weeks after transfer, we observed a strongly reduced cellularity in the thymus, LNs, and spleen of the CD45.1^+^ recipient mice receiving CD45.2^+^ *Vav*-Cre*Nfatc1*^fl/fl^ splenocytes, indicating defects in lymphopoiesis (Figure 3a). An evaluation of the CD45.2^+^ donor-derived cells in these organs showed a strongly reduced reconstitution capacity of *Vav*-Cre*Nfatc1*^fl/fl^ splenocytes as compared with *Nfatc1*^fl/fl^ cells (Figure 3b). Further analysis revealed that *Vav*-Cre*Nfatc1*^fl/fl^ splenocytes gave rise to very few CD4^+^ and CD8^+^ T cells in the NFATc1-sufficient recipient mice (Figure 3c). Similarly, the analysis of B cell populations in the BM and spleen of the recipient mice revealed a much lower reconstitution of IgM^+^ and IgD^+^ B cells in the case of *Vav*-Cre*Nfatc1*^fl/fl^ donors as compared with *Nfatc1*^fl/fl^ donors (Figure 3d). These defects in T and B cell development recapitulate the severe lymphopenia in *Vav*-Cre*Nfatc1*^fl/fl^ mice we have reported recently and suggest that HSPC-specific defects also contribute to this lymphopenia in addition to other signaling defects.

### 3.4. NFATc1 Deficiency Severely Impairs Myelopoiesis

**Figure 4:**
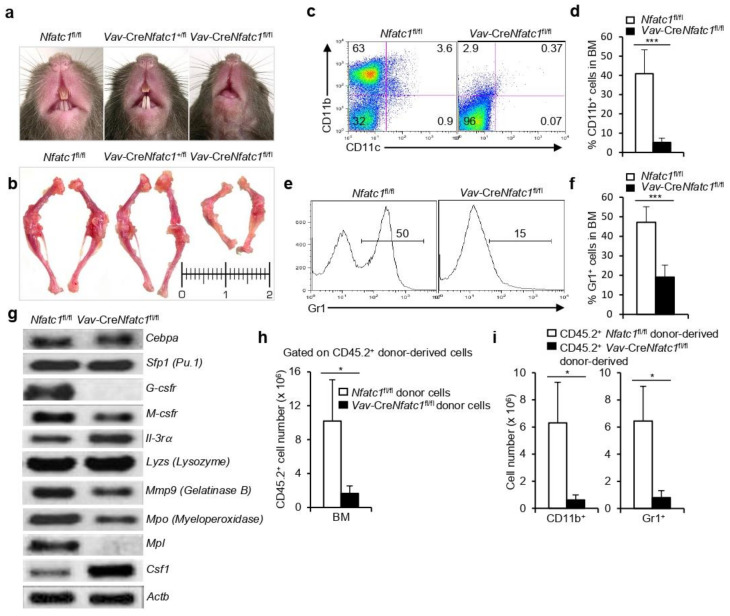
**Severely impaired myelopoiesis in *Vav*-Cre*Nfatc1*^fl/fl^ mice**: (**a**) Photographs of *Vav*-Cre*Nfatc1*^fl/fl^ mice showing lack of tooth eruption as compared with littermate control mice; (**b**) photographs of the bones from the hind limbs (femur and tibia) of indicated mice; (**c**) distribution of CD11b^+^ and CD11c^+^ cells in the BM of *Vav*-Cre*Nfatc1*^fl/fl^ mice as compared with littermate *Nfatc1*^fl/fl^ mice, as revealed by flow cytometry; (**d**) quantification of percent CD11b^+^ cells distribution in the BM of *Vav*-Cre*Nfatc1*^fl/fl^ mice (*n* = 4) and *Nfatc1*^fl/fl^ (*n* = 5) littermates; (**e**) BM distribution of Gr1^+^ cells in *Vav*-Cre*Nfatc1*^fl/fl^ mice and *Nfatc1*^fl/fl^ littermates; (**f**) quantification of Gr1^+^ cells distribution in the BM of indicated mice (*Nfatc1*^fl/fl^, *n* = 11 and *Vav*-Cre*Nfatc1*^fl/fl^, *n* = 6); (**g**) myelopoiesis-related gene expression in BM cells from *Vav*-Cre*Nfatc1*^fl/fl^ mice as compared with *Nfatc1*^fl/fl^ mice; (**h**) CD45.2^+^ *Nfatc1*^fl/fl^ or *Vav*-Cre*Nfatc1*^fl/fl^ donor-derived cell numbers in the BM (* *p* = 0.0405) of CD45.1^+^ recipient mice, 11 weeks post cell transfer; (**i**) number of CD45.2^+^ *Nfatc1*^fl/fl^ or *Vav*-Cre*Nfatc1*^fl/fl^ donor-derived CD11b^+^ and Gr1^+^ cells in the BM (* *p* = 0.0307 and * *p* = 0.0200, respectively) of CD45.1^+^ recipient mice. Littermate *Nfatc1*^fl/fl^ and *Vav*-Cre*Nfatc1*^fl/fl^ mice used in the experiments were 3 weeks old. Data in (**a**–**g**) are representative of more than three independent experiments, and in (**h**,**i**) are representative of two independent experiments. Numbers inside each dot plot or histogram represent percent respective populations. Data in (**d**) (*** *p* = 0.0009), (**f**) (*** *p* < 0.0001), and (**h**,**i**) are presented as mean ± s.d., unpaired *t*-test.

Owing to the impaired lymphopoiesis, we investigated whether other branches of hematopoiesis were also affected due to lack of NFATc1 activity in *Vav*-Cre*Nfatc1*^fl/fl^ mice. Strikingly, the phenotypic analysis revealed a lack of teeth eruption in *Vav*-Cre*Nfatc1*^fl/fl^ mice as compared with littermate controls (Figure 4a). This was consistent in all *Vav*-Cre*Nfatc1*^fl/fl^ mice analyzed, suggesting a possible defect in myeloid cell development. The complete absence of teeth in *Vav*-Cre*Nfatc1*^fl/fl^ mice as compared with the normal teeth eruption in *Vav*-Cre*Nfatc1*^+/fl^ mice indicates an absolute requirement of NFATc1 activity in this process. In addition to the lack of teeth eruption, bone formation in *Vav*-Cre*Nfatc1*^fl/fl^ mice was also defective, as bones from the hind limbs were short and more solid as compared with the littermate control mice (Figure 4b), indicating osteopetrosis. The role of NFATc1 in osteoblast differentiation and bone formation has been reported [26,27], and their involvement in bone-resorbing osteoclast development has also been suggested [28,29]. Osteoclasts differentiate from the CD11b^+^ myeloid cells. Interestingly, the flow cytometry analysis to explore whether the teeth eruption problem was due to a defect in osteoblast or osteoclast activity in *Vav*-Cre*Nfatc1*^fl/fl^ mice, revealed a complete lack of CD11b^+^ cells in the BM (Figure 4c,d). This suggested that the defect in *Vav*-Cre*Nfatc1*^fl/fl^ mice was in the osteoclast differentiation. Combined with the loss of CD11b^+^ cells, severely reduced Gr1^+^ cells in *Vav*-Cre*Nfatc1*^fl/fl^ mice (Figure 4e,f) suggested a strong defect in myelopoiesis in the absence of NFATc1 activity. The gene expression analysis showed downregulated expression in *Vav*-Cre*Nfatc1*^fl/fl^ mice of several genes responsible for myeloid cell development and function (Figure 4g). Although there was an increase in the expression of the colony stimulating factor (*Csf1*) gene, suppressed expression of the receptors (*G-Csfr* and *M-Csfr*) most likely contributed to the lack of myeloid cells in *Vav*-Cre*Nfatc1*^fl/fl^ mice (Figure 4g). The defects in myelopoiesis in *Vav*-Cre*Nfatc1*^fl/fl^ mice were appropriately reflected in our adoptive transfer experiments, where 11 weeks post transfer, analysis of the CD45.2^+^ donor-derived cells revealed a strong decrease in the myeloid reconstitution (CD11b^+^ and Gr1^+^ cells) potential of *Vav*-Cre*Nfatc1*^fl/fl^ HSCs (Figure 4h,i). The impaired myelopoiesis in *Vav*-Cre*Nfatc1*^fl/fl^ mice was specific to NFATc1 deficiency, as *Nfatc2*^−/−^ or *Nfatc3*^−/−^ mice did not show these abnormalities in myelopoiesis (Supplementary Figure S3a–d). In addition to the lack of myeloid cells, we recently reported an enhanced production of Ter119^+^ erythroid cells in *Vav*-Cre*Nfatc1*^fl/fl^ mice, which suggested that the process of erythropoiesis was also dysregulated in the absence of NFATc1 activity [20]. Taken together, the lack of NFATc1 activity in the hematopoietic system severely impairs development and differentiation of multiple hematopoietic lineages.

### 3.5. NFATc1 Activity Is Indispensable for Normal Hematopoiesis

**Figure 5:**
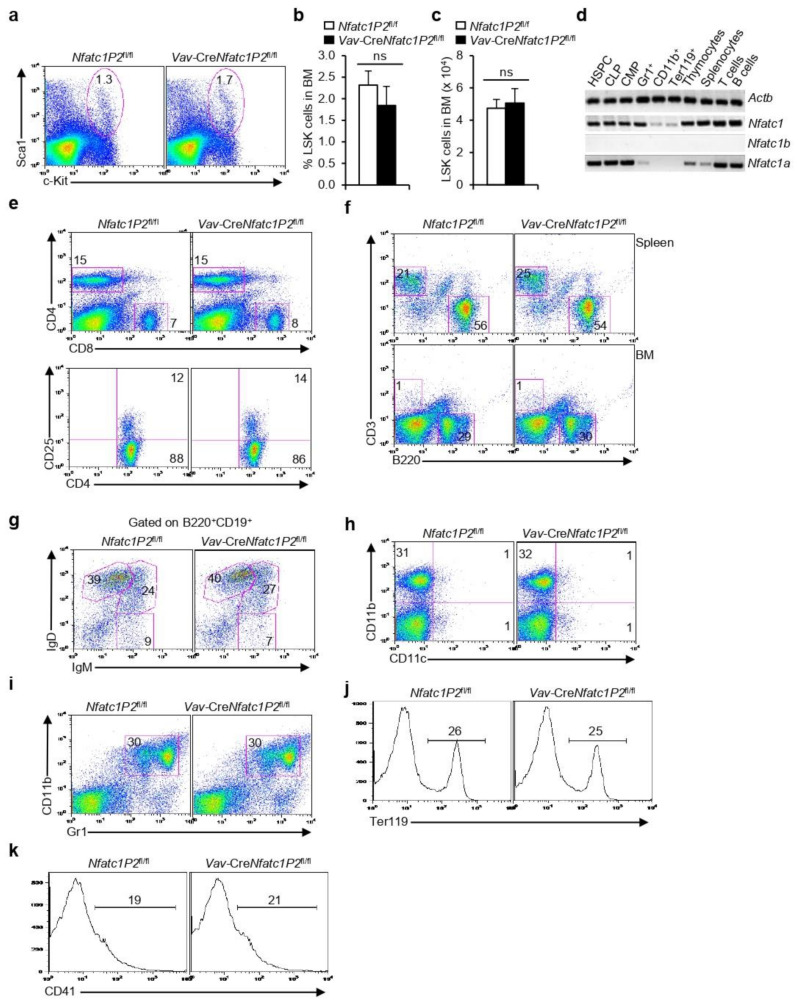
**Normal hematopoiesis in *Vav*-Cre*Nfatc1P2*^fl/fl^ mice**: (**a**) Distribution of LSK cells in the BM of *Vav*-Cre*Nfatc1P2*^fl/fl^ mice as compared with *Nfatc1P2*^fl/fl^ mice, as revealed by flow cytometry; (**b**) quantification of percent LSK cells distribution in the BM of *Vav*-Cre*Nfatc1P2*^fl/fl^ (*n* = 4) and *Nfatc1P2*^fl/fl^ (*n* = 5) mice; (**c**) absolute LSK cell numbers in the BM of *Vav*-Cre*Nfatc1P2*^fl/fl^ (*n* = 6) mice as compared with littermate *Nfatc1P2*^fl/fl^ (*n* = 11) mice; (**d**) RT-PCR analysis reveals expression levels of total *Nfatc1*, and the *Nfatc1a* and *Nfatc1b* isoforms in sorted populations as indicated from the *Vav*-Cre*Nfatc1P2*^fl/fl^ mice BM; (**e**) distribution of CD4^+^, CD8^+^, and CD4^+^CD25^+^ T cells in the spleen of *Vav*-Cre*Nfatc1P2*^fl/fl^ mice as compared with *Nfatc1P2*^fl/fl^ mice, as revealed by flow cytometry; (**f**) distribution of CD3^+^ and B220^+^ cells in the BM and spleen of indicated mice; (**g**) IgM^+^ and IgD^+^ B cells distribution gated on B220^+^CD19^+^ cells in the spleen of indicated mice; (**h**–**k**) BM distribution of CD11b^+^ and CD11c^+^ cells (**h**), CD11b^+^ and Gr1^+^ cells (**i**), Ter119^+^ cells (**j**), and CD41^+^ cells (**k**), in *Vav*-Cre*Nfatc1P2*^fl/fl^ mice as compared with *Nfatc1P2*^fl/fl^ mice, as revealed by flow cytometry. Data are representative of three independent experiments. Numbers inside each FACS plot represent percent respective populations. Data in (**b**,**c**) are presented as mean ± s.d., ns = not significant, unpaired *t*-test.

Ssince the NFATc1 in hematopoietic stem and progenitor cells was mainly of the P2 promoter-derived NFATc1β isoforms, next, we investigated if NFATc1β was critical for HSPC development and hematopoiesis. To check if HSPC development was affected in the absence of NFATc1β, we analyzed *Vav*-Cre*Nfatc1P2*^fl/fl^ mice in which *Nfatc1* P2 promoter activity was ablated in the hematopoietic system [18]. We observed normal levels of HSPCs in *Vav*-Cre*Nfatc1P2*^fl/fl^ mice as compared with littermate control mice (Figure 5a–c). This was quite intriguing considering the grossly impaired HSPC population in *Vav*-Cre*Nfatc1*^fl/fl^ mice (Figure 2e–g). The RT-PCR analysis revealed a complete absence of P2 promoter activity in *Vav*-Cre*Nfatc1P2*^fl/fl^ mice (Figure 5d). However, in the absence of P2 activity, a strong P1 promoter-derived *Nfatc1a* isoform expression was detected in HSPCs and in all hematopoietic lineage cells (Figure 5d), which suggested that NFATc1 activity was present in these cells.

As a result of normal HSPC numbers in *Vav*-Cre*Nfatc1P2*^fl/fl^ mice, the development of lymphoid, myeloid, and erythroid cells remained unaffected. The distribution of various T and B cell populations in peripheral lymphoid organs was comparable to that of littermate control mice (Figure 5e–g). Interestingly, the stark defects in myelopoiesis in terms of the development of CD11b^+^ and Gr1^+^ cells in *Vav*-Cre*Nfatc1*^fl/fl^ mice were not observed in *Vav*-Cre*Nfatc1P2*^fl/fl^ mice (Figure 5h,i). In addition, the alterations in erythropoiesis in the absence of NFATc1 activity were rescued in *Vav*-Cre*Nfatc1P2*^fl/fl^ mice (Figure 5j,k). As a result of normal hematopoiesis, *Vav*-Cre*Nfatc1P2*^fl/fl^ mice did not exhibit any of the defects observed in the case of *Vav*-Cre*Nfatc1*^fl/fl^ mice (Figure 2) and had a normal lifespan. Altogether, the analysis of *Vav*-Cre*Nfatc1P2*^fl/fl^ mice established that NFATc1 activity irrespective of the α or β isoforms was indispensable for HSPC maintenance and normal hematopoiesis.

### 3.6. Increase in NFATc1 Activity Impairs Hematopoiesis

**Figure 6:**
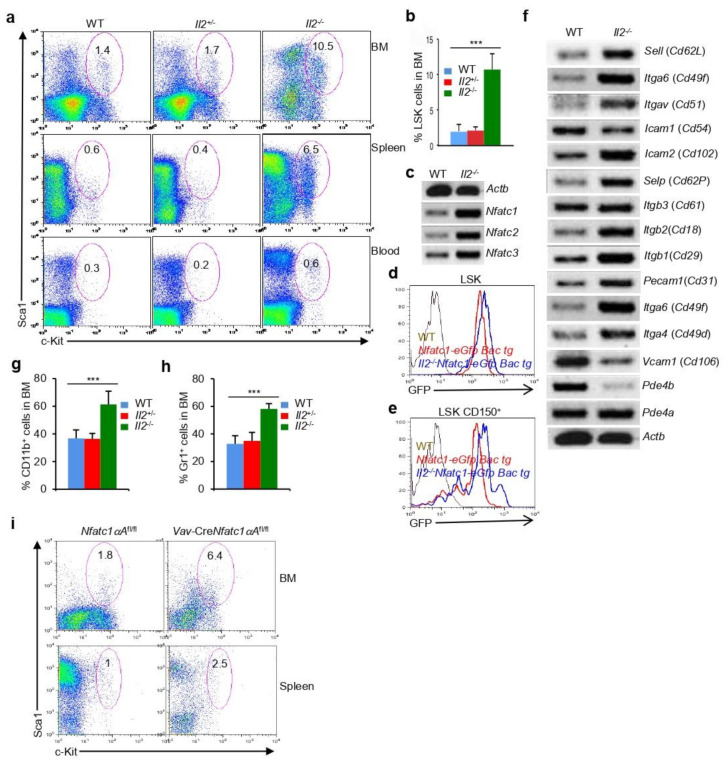
**Enhanced NFAT activity impairs hematopoiesis**: (**a**) Distribution of LSK cells in the BM, spleen, and blood from *Il2*^−/−^ mice as compared with littermate controls. The number inside each plot represents percent LSK cells; (**b**) quantification of percent LSK cells in the BM of WT (*n* = 6), *Il2*^+/−^ (*n* = 9), and *Il2*^−/−^ (*n* = 12) mice; (**c**) levels of *Nfatc1*, *Nfatc2*, and *Nfatc3* expression in sorted BM LSK cells from *Il2*^−/−^ mice as compared with WT littermates, as revealed by RT-PCR; (**d**) GFP expression representing NFATc1 levels in BM LSK cells from *Il2*^−/−^*Nfatc1-eGfp-Bac* tg reporter mice as compared with *Nfatc1-eGfp-Bac* tg mice; (**e**) GFP expression representing NFATc1 levels in BM LSKCD150^+^ HSCs from *Il2*^−/−^*Nfatc1-eGfp-Bac* tg reporter mice as compared with *Nfatc1-eGfp-Bac* tg mice; (**f**) RT-PCR analysis of the expression levels of various integrin in sorted BM LSK cells from WT and *Il2*^−/−^ mice; (**g**) distribution of CD11b^+^ cells in the BM of *Il2*^−/−^ mice as compared with littermate controls; (**h**) Gr1^+^ cells distribution in the BM of *Il2*^−/−^ mice as compared to littermate controls. (**i**) Distribution of LSK cells in the BM, and spleen from *Vav-CreNfatc1aA^fl/f^* mice compared with littermate controls. Number inside each plot represents percent LSK cells. In (**g**,**h**), *n* = 7 (WT), 8 (*Il2*^+/−^), and 7 (*Il2*^−/−^) mice. Data in (**a**,**b**,**g**,**h**) are representative of more than three experiments and, in (**c**–**f**,**i**), data represent two independent experiments. Data in (**b**,**g**,**h**) are presented as mean ± s.d., *** *p* < 0.0001, one-way ANOVA.

Next, we investigated if the loss of NFATc1 activity resulted in a loss of HSPCs and a defective hematopoiesis; an increase in NFATc1 activity would probably augment HSPC development and differentiation of various lineages. In *Il2*^−/−^ mice, the frequency of HSPCs is increased several times as compared with that in littermate control mice [30]. This holds true in the BM, spleen, and in the blood from all *Il2*^−/−^ mice irrespective of gender (Figure 6a,b). We hypothesized that most likely there is an increase in NFAT activity in *Il2*^−/−^ HSPCs, which facilitates enhanced levels of HSPC development. The RT-PCR analysis revealed a strongly increased *Nfat* expression in *Il2*^−/−^ HSPCs as compared with that in WT mice (Figure 6c). In addition, the analysis of *Il2*^−/−^*Nfatc1-eGfp-Bac* tg reporter mice further demonstrated increased NFATc1 levels in HSPCs and HSCs as compared with control cells (Figure 6d,e). Increased NFATc1 levels were also observed in the c-Kit^+^ and Sca1^+^ cells from *Il2*^−/−^*Nfatc1-eGfp-Bac* tg reporter mice (Supplementary Figure S4a). We previously showed that, in *Il2*^−/−^ mice, there is an increase in LT-HSCs and a corresponding decrease in the ST-HSCs [30], which was exactly the opposite of what we have observed in the case of *Vav*-Cre*Nfatc1*^fl/fl^ mice (Figure 2h). This further suggests that alterations in the level of NFATc1 activity will certainly have an adverse influence on HSPC maintenance.

The increased *Nfat* expression is most likely due to enhanced integrin-cAMP signaling in the *Il2*^−/−^ HSPCs. HSPCs generally express several integrins (Supplementary Figure S4b), and our analysis revealed that *Il2*^−/−^ HSPCs express much higher levels of various integrins as compared with WT HSPCs (Figure 6f). Integrin signaling has been reported to induce cAMP signaling [31,32,33], and recently, we showed that integrin-cAMP signaling was a critical regulator of *Nfatc1* expression [18]. Complementing the increased integrin expression, we observed decreased phosphodiesterase (*Pde4b*) expression in *Il2*^−/−^ HSPCs, which further supported the enhanced cAMP activity in these cells as compared with WT HSPCs (Figure 6f).

We analyzed *Il2*^−/−^ mice for various lineage-positive cells to explore if enhanced HSC numbers in *Il2*^−/−^ mice also enhanced hematopoiesis. Interestingly, in *Il2*^−/−^ mice, we observed strongly increased myeloid cell populations as compared with littermate WT mice. CD11b^+^ and Gr1^+^ myeloid cell differentiation was increased in *Il2*^−/−^ mice (Figure 6g,h), which was in stark contrast to the situation in *Vav*-Cre*Nfatc1*^fl/fl^ mice. We previously showed that there was an increased expression of genes responsible for myelopoiesis in *Il2*^−/−^ mice [30], which was suppressed in *Vav*-Cre*Nfatc1*^fl/fl^ mice (*Csf1r*, *Csf3r*, and *Mpl*, Figure 4g). In addition, contrary to the enhanced Ter119^+^ erythroid cell differentiation in *Vav*-Cre*Nfatc1*^fl/fl^ mice, differentiation of these cells in *Il2*^−/−^ mice was drastically reduced, which we reported earlier [20]. The enhanced T cell population in *Il2*^−/−^ mice was also in contrast to the lymphopenic situation in *Vav*-Cre*Nfatc1*^fl/fl^ mice. Taken together, hematopoietic phenotype in *Il2*^−/−^ mice with an increased NFATc1 activity in HSPCs completely reverses the phenotype in NFATc1-deficient *Vav*-Cre*Nfatc1*^fl/fl^ mice. Although the hematopoiesis in *Il2*^−/−^ mice is also severely dysregulated due to additional factors, it clearly suggests that alteration in NFATc1 activity is certainly detrimental for HSPC maintenance and differentiation of multiple hematopoietic lineages.

Increased HSPC population due to enhanced NFATc1 activity was also observed in *Vav-creNfatc1αA*^fl/fl^ mice (Figure 6i), in which NFATc1 activity in the hematopoietic cells was enhanced due to the expression of a constitutively active form of NFATc1 in a Cre-dependent manner [18].

## 4. Discussion

The roles of NFAT factors in lymphocyte development and function have been studied in detail [12,13,15,34,35,36]. However, so far, the influence of NFAT proteins in HSC maintenance has not been properly investigated. A previous report suggested the involvement of NFAT in the maintenance of LT- and ST-HSCs [37], and a more recent report suggested suppression of NFAT activity was essential to maintain lymphoid-primed HSCs [38]. However, in both of these studies, NFAT activity was measured as a readout for an increase in intracellular calcium ion. In addition, the model systems used in these studies ruled out the the possibility that the observed effects on HSCs were solely because of altered NFAT activity. Adopting multiple approaches, we showed that NFAT family TFs were expressed in HSPCs, which might play critical roles in their maintenance and in the differentiation of various lineage-positive cells. Our observations show that, among the NFAT family members, NFATc1 activity plays a key role in HSPC maintenance and is indispensable for normal hematopoiesis. NFATc1 activity in maintaining the quiescence of skin stem cells has been reported [39], but its role in HSPC maintenance has not yet been explored in detail. The fact that HSPC phenotype and the hematopoiesis in *Vav*-Cre*Nfatc1*^fl/fl^ mice were severely affected resulting in a drastic reduction in multiple lineage-positive cells, suggests NFATc1 activity is intimately associated with hematopoiesis, and thereby the survival of an organism. Accordingly, *Vav*-Cre*Nfatc1*^fl/fl^ mice lacking NFATc1 activity in the hematopoietic system die at a very early age (Figure 2k). This severe phenotype in HSPC maintenance and hematopoiesis in *Vav*-Cre*Nfatc1*^fl/fl^ mice occurs despite them having intact NFATc2 and NFATc3 activity. *Nfatc2*^−/−^, *Nfatc3*^−/−^, or *Nfatc2*^−/−^*Nfatc3*^−/−^ mice did not show the hematopoietic phenotype that we observed in *Vav*-Cre*Nfatc1*^fl/fl^ mice, suggested that they were unable to substitute for NFATc1 activity in HSPC maintenance and hematopoiesis.

NFATc1 expression in HSCs, and also in all subsequent stages that are derived from them and in the lineage-positive cells, is an interesting observation. NFATc1 activity in various hematopoietic cells have been reported [15,16,40,41,42]. We showed that NFATc1 was also expressed in the progenitor populations of all hematopoietic lineages (Figure 2m), which attests that, at every stage, NFATc1 plays an important role that guides normal hematopoiesis. The early death of *Vav*-Cre*Nfatc1*^fl/fl^ mice also shows how critical NFATc1 activity is for the development of hematopoietic cells. Recently, we reported severe defects in T and B cell development in *Vav*-Cre*Nfatc1*^fl/fl^ mice [14,18,19]. Although these defects were characterized at a later stage during development, they suggested that, most likely, there were additional defects at an earlier stage, which exacerbated the situation at the later stages. Our findings of defective HSPC maintenance in *Vav*-Cre*Nfatc1*^fl/fl^ mice support this notion. In fact, the low expression of *Cd150* and a high expression of *Flk2* in *Vav*-Cre*Nfatc1*^fl/fl^ HSCs (Figure 2h) suggests a paucity of LSKCD150^+^Flk2^−^ LT-HSCs and an increase in the LSKCD150^−^Flk2^+^ ST-HSCs in the absence of NFATc1 activity. This could be a major contributor to the defects at the subsequent stages of hematopoiesis in these mice. It is quite intriguing that, despite stronger expression of many genes that are involved in maintaining HSC quiescence, survival, and proliferation (Figure 2i), there is such a strong impairment in the HSPC numbers in *Vav*-Cre*Nfatc1*^fl/fl^ mice. HSPCs in the absence of NFATc1 activity were also functionally defective as in adoptive transfer experiments, *Vav*-Cre*Nfatc1*^fl/fl^ HSPCs failed to efficiently repopulate the hematopoietic system of the recipient mice (Figure 3 and Figure 4h,i).

We have previously shown severe defects in lymphopoiesis and erythropoiesis due to altered NFATc1 activity(14, 18–20). In addition, lack of or enhanced NFATc1 activity have been shown to be involved in many pathologies associated with the hematopoietic system [43,44]. We show that, as compared with the littermate control mice, *Vav*-Cre*Nfatc1*^fl/fl^ mice completely lack teeth eruption (Figure 4a). NFATc1 activity in bone formation is well documented as it plays critical roles in osteoblast and osteoclast differentiation [26,27,28,29]. However, in *Vav*-Cre*Nfatc1*^fl/fl^ mice, the lack of teeth eruption is unlikely to be due to a defect in osteoblast activity, as the bones in these mice are well developed although not normal as compared with the control mice (Figure 4b). Rather, it could be due to a lack of activity in the case of bone resorbing osteoclast cells, which leads to the failure in teeth eruption. Osteoclasts are myeloid lineage cells that are differentiated from the CD11b^+^ precursor cells [45,46]. Our observation regarding the complete lack of CD11b^+^ cells in *Vav*-Cre*Nfatc1*^fl/fl^ mice provides evidence that NFATc1 activity is indispensable for their development, and thereby in osteoclast differentiation. Thus, due to lack of osteoclast activity, teeth failed to erupt in the jawbones when NFATc1 activity was absent. In addition to the lack of CD11b^+^ cells, *Vav*-Cre*Nfatc1*^fl/fl^ mice also had a severe problem in the development of Gr1^+^ cells, which gave rise to granulocytes such as neutrophils [47,48]. The Gr1^+^ cell number in the BM of *Vav*-Cre*Nfatc1*^fl/fl^ mice was very low as compared with the littermate control mice (Figure 4e,f). These developmental defects were due to impaired expression in these mice of several genes related to the myeloid cell development (Figure 4g). NFATc1-mediated defects in DC development have been reported previously [17,42,49]. The defects in the development of various myeloid cells that we have shown in *Vav*-Cre*Nfatc1*^fl/fl^ mice suggest that NFATc1 activity is not only involved in lymphopoiesis or erythropoiesis, but also critically regulates myelopoiesis. Thus, NFATc1 is a key regulator of the differentiation of multiple hematopoietic lineage cells.

The differential expression of two promoters regulating *Nfatc1* expression provides further evidence that NFATc1 activity is vital for HSPC maintenance and normal hematopoiesis. The prevalence of P2 promoter-derived *Nfatc1b* expression in hematopoietic stem and progenitor cells as well as in lineage-positive cells (Figure 2m) suggests that a constitutive NFATc1 activity is required for normal hematopoiesis. The substitution of NFATc1β activity in hematopoiesis by P1 promoter-derived NFATc1α in *Vav*-Cre*Nfatc1P2*^fl/fl^ mice further underlines the indispensability of NFATc1 activity, irrespective of the isoforms in HSPC maintenance and lineage differentiation (Figure 5). Additionally, a reverse phenotype to that of *Vav*-Cre*Nfatc1*^fl/fl^ mice in HSPC maintenance and in the differentiation of various lineage-positive cells, in *Il2*^−/−^ mice, reveals the association of NFATc1 activity in hematopoiesis. The increase in HSPC numbers, the accumulation of LT-HSCs, hugely increased numbers of CD11b^+^ and Gr1^+^ cells, as well as the lack of Ter119^+^ erythroid cells in *Il2*^−/−^ mice (Figure 6 and [20,30,50]) were completely in contrast to the phenotype we observed in *Vav*-Cre*Nfatc1*^fl/fl^ mice. In addition, due to enhanced NFATc1 activity in *Vav-creNfatc1αA*^fl/fl^ mice, the HSPC population was significantly increased (Figure 6i), suggesting an essential role of NFATc1 in HSPC physiology. The contrasting hematopoietic phenotype due to the lack of (*Vav*-Cre*Nfatc1*^fl/fl^ mice), or increased (*Il2*^−/−^ and *Vav-creNfatc1αA*^fl/fl^ mice), NFATc1 activity in the HSPCs underlines the essentiality of an optimal level of NFATc1 activity in facilitating hematopoiesis, and thereby the survival of an organism. Altogether, our study delineates a novel parameter in terms of NFATc1 activity in HSPC maintenance and in lineage differentiation, manipulation of which could help in multiple clinical conditions of dysregulated hematopoiesis in humans.

## Supplementary Materials

The following supporting information can be downloaded at: https://www.mdpi.com/article/10.3390/cells11132012/s1, Figure S1: Flow cytometry profile showing the gating strategy for identification of BM cell populations. Figure S2: GFP levels representing NFATc1 expression in myeloid and erythroid cell populations. Figure S3: Distribution of CD11b^+^ and CD11c^+^ cells in the BM of *Nfatc2*^−/−^ and *Nfatc*^−/−^ mice. Figure S4: NFATc1 levels in BM Lin^−^Sca1^+^c-Kit^−^ and Lin^−^Sca1^−^c-Kit^+^ cells from *Il2*^−/−^*Nfatc1-eGfp-Bac* tg reporter mice. Table S1: List of RT-PCR primers.
