# Supplementary material for: Hematopoietic Stem and Progenitor Cell Maintenance and Multiple Lineage Differentiation Is an Integral Function of NFATc1
Source: Cells. 2022 Jun 23;11(13):2012. doi: 10.3390/cells11132012 (PMC9265824; doi:10.3390/cells11132012)
Supplement: Supplementary file 1 [file cells-11-02012-s001.zip › Patra_Supplementary Materials.pdf]

SUPPLEMENTARY MATERIALS

Hematopoietic stem and progenitor cell maintenance and multiple lineage differentiation is an integral function of NFATc1

Carlotta Barahona de Brito[1,2], **Stefan Klein-Hessling**[3], **Edgar Serfling**[3,4] **& Amiya Kumar Patra**[1]

[1]Peninsula Medical School, University of Plymouth, Plymouth PL6 8BU, UK.

[2]Department of Biology and Biochemistry, University of Bath, Bath BA2 7AX, UK.

[3]Department of Molecular Pathology, Institute of Pathology, University of Würzburg, Josef Schneider-Str. 2, 97080 Würzburg, Germany, and

[4]Comprehensive Cancer Center Mainfranken, University of Würzburg, Josef Schneider-Str. 6, 97080 Würzburg, Germany.

Key words: Hematopoiesis, HSC, Lineage differentiation, and NFATc1

Running title: NFATc1 regulates hematopoiesis

Total number of figures: 6

Reprint requests and page proofs should be addressed to:

Dr. Amiya K. Patra
Peninsula Medical School
University of Plymouth,
Plymouth Science Park, Research Way
Plymouth PL6 8BU
United Kingdom.

Tel.: + 44 1752 437433
E. mail: amiya.patra@plymouth.ac.uk

SUPPLEMENTARY FIGURE LEGENDS

Supplementary Figure 1. (a) Flow cytometry profile showing the gating strategy for identification of BM Lin$^-$Sca1$^+$c-Kit$^+$ (LSK), Lin$^-$Sca1$^+$c-Kit- (Sca1$^+$), Lin$^-$Sca1$^-$c-Kit$^+$ (c-Kit$^+$) and Lin$^-$Sca1$^-$c-Kit$^-$ (L$^-$S$^-$K$^-$) cells from WT mice. (**b**) Immunofluorescence analysis for nuclear NFATc1 levels in sorted LSK, Sca1$^+$, and c-Kit$^+$ cells from WT mice. Co-staining with DAPI confirms nuclear NFATc1. Scale bar, 10 µm. Data are representative of three independent experiments.

Supplementary Figure 2. GFP levels representing NFATc1 expression in myeloid and erythroid cell populations from spleen and BM of *Nfatc1-eGfp-Bac* tg reporter mice compared to WT mice. Numbers inside each histogram represent mean fluorescent intensity (MFI). Data are representative of three independent experiments.

Supplementary Figure 3. (a) Flow cytometry profiles showing the distribution of CD11b$^+$ and CD11c$^+$ cells in the BM of *Nfatc2*$^{-/-}$ and *Nfatc3*$^{-/-}$ mice compared to littermate WT mice. (**b**) Quantification of percent CD11b$^+$ cells distribution in the BM of *Nfatc2*$^{-/-}$, *Nfatc3*$^{-/-}$ mice (n = 3 each) and WT (n = 5) littermates. (**c**) Distribution of CD11b$^+$ and Gr1$^+$ cells in the BM of *Nfatc2*$^{-/-}$ and *Nfatc3*$^{-/-}$ mice compared to littermate WT mice as revealed by flow cytometry. (**d**) Quantification of percent Gr1$^+$ cells distribution in the BM of *Nfatc2*$^{-/-}$, *Nfatc3*$^{-/-}$ mice (n = 3 each) and WT (n = 5) littermates. Numbers inside each dot plot represent percent respective populations (ns = not significant; one-way ANOVA).

Supplementary Figure 4. (a) Flow cytometry analysis of GFP expression representing NFATc1 levels in BM Lin$^-$Sca1$^+$c-Kit$^-$ and Lin$^-$Sca1$^-$c-Kit$^+$ cells from *Il2*$^{-/-}$*Nfatc1-eGfp-Bac* tg reporter mice compared to *Nfatc1-eGfp-Bac* tg mice. Wild-type mice were used as

negative control. (**b**) Analysis of various integrins (CD62L, CD11a, CD18 and CD49d) expression on HSCs from WT mice.

Supplementary Table S1

LIST OF RT-PCR PRIMERS

Gene	Primer Sequence	Product Size
Actb	For: 5'- CCAGGTCATCACTATTGGCAAGGA -3'<br>Rev: 5'- GAGCAGTAATCTCCTTCTGCATCC -3'	223 bp
Cebpa	For: 5'- CGCTGGTGATCAAACAAGAG -3'<br>Rev: 5'- TCACTGGTCAACTCCAGCAC -3'	499 bp
cMyc	For: 5'- CATCCTGTCCATTCAAGC -3'<br>Rev: 5'- TAATTCCAGCGCATCAGT -3'	200 bp
Csf1	For: 5'- AGTCTGTCTTCCACCTGCTG -3'<br>Rev: 5'- TGGTAGTGGTAGGCCACATT -3'	299 bp
Egr2	For: 5'- CTCCCATCTCTGCACCTAGA -3'<br>Rev: 5'- ATAAGGAGGAGGAGGTGGTG -3'	284 bp
Flk2	For: 5'- GACAAATCTCCCAATTGCAC -3'<br>Rev: 5'- GTGGCAGATCAACACAATGA -3'	291 bp
Fog1	For: 5'- ATCCAGTCAGGGTGAAGACA -3'<br>Rev: 5'- AGGTGATCTCGCACTCAAAG -3'	291 bp
Csf3r (Gcsfr)	For: 5'- AACTACACCCAGGCCTTCCT -3'<br>Rev: 5'- GAGCTCAAACTGGTCCTTGC -3'	606 bp
Mmp9 (Gelatinase B)	For: 5'- CGTCGTGATCCCCACTTACT -3'<br>Rev: 5'- AGAGTACTGCTTGCCCAGGA -3'	433 bp
Gfi1	For: 5'- GAGCTTCAAGAGGTCATCCA -3'<br>Rev: 5'- AGTCCATGCTGAGTCTCTCG -3'	306 bp
Hax1	For: 5'- GATGACGACGATGATGATGA -3'<br>Rev: 5'- CTGGGTTGGTGACTATCTGG -3'	330 bp
HoxA9	For: 5'- TCACCAACCAAACACAACAG-3'<br>Rev: 5'- CACAATTAATCACGCCATCA-3'	299 bp
Icam1	For: 5'-CCAAGAAACGCTGACTTCAT-3'<br>Rev: 5'- CGACCCTTATGAGAAAAGCA-3'	327 bp
Icam2	For: 5'- GAAGCCACAGAGTCTTGGAA-3'<br>Rev: 5'- TCAGTGTGACTTGAGCTGGA-3'	244 bp
Il3ra	For: 5'-GCCCTGCATGGACAACACT-3'<br>Rev: 5'-GCACCGTAGCCACTGAAGTCA-3'	423 bp
Itga4	For: 5'- AAGCCAGCGTTCATATTCAG-3'<br>Rev: 5'- ATCCAGCCTTCCACATAACA-3'	277 bp
Itga6	For: 5'-TGAGGTGTGTGAACATCAGG-3'<br>Rev: 5'-TAGAGCCAGCATCAGAATCC-3'	301 bp
Itgav	For: 5'- GGAGAACCAGAACCATTCCT-3'<br>Rev: 5'- TTGCTCTTCTTGAGGTGGTC-3'	272 bp
Itgb1	For: 5'- AAGACATGGACGCTTACTGC-3'<br>Rev: 5'- ATGGACCAGTGTCCAAAGAA-3'	267 bp
Itgb2	For: 5'- TAATGCAAGTTGCTGCATGT-3'<br>Rev: 5'- GCTGGAGTCGTCAGACAGTT-3'	332 bp

Itgb3	For: 5'-ATACCAGGGAGGACCTTCAG-3'<br>Rev: 5'-TCCTTCCCTGCTAGTTTCCT-3'	257bp
Lef1	For: 5'- CCAGACTGTCTCCACAGCTT-3'<br>Rev: 5'- TGACAGGTCAGCACAGAAGA-3'	288 bp
Lyzs (Lysozyme)	For: 5'- ACTGCTCAGGCCAAGGTCTA-3'<br>Rev: 5'- GCCCTGTTTCTGCTGAAGTC-3'	524 bp
Csf1r (Mcsfr)	For: 5'-ATGAGTCCCTCTTCACTCCG-3'<br>Rev: 5'-ACCTTCAGCACTGCATCTTC-3'	306 bp
Meis1	For: 5'- AATGCCTATCGATTTGGTGA-3'<br>Rev: 5'- CCTTATCAGGGTCATCATCG-3'	266 bp
Mpl	For: 5'- GAAATCTGCCTGCTGTGACT-3'<br>Rev: 5'- GAACCAGGAAGGAAGGTGAT-3'	254 bp
(Mpo) Myeloperoxidase	For: 5'-ATGCAGTGGGGACAGTTTCTG-3'<br>Rev: 5'-GTCGTTGTAAGATCGGTACTG-3'	696 bp
Nfatc1	For: 5'-GACTTCGATTTCCTCTTCGAGTTC-3'<br>Rev: 5'-CTCGATTCTCGGACTCTCCAG-3'	297 bp
Nfatc1a	For: 5'- GGGAGCGGAGAAACTTTGC -3'<br>Rev: 5'- GATCTCGATTCTCGGACTCTCC -3'	319 bp
Nfatc1b	For: 5'-CGACTTCGATTTCCTCTTCGAG -3'<br>Rev: 5'- GATCTCGATTCTCGGACTCTCC-3'	311 bp
Nfatc2	For: 5'-GGGTTCGGTGAGTGACAGTT-3'<br>Rev: 5'-CTCCTTGGCTGTTTGGGATA-3'	371 bp
Nfatc3	For: 5'-CCGATGACTACTGCAAACTGTGG-3'<br>Rev: 5'-TTTGAATACTTGGGCACTCAAAGG-3'	343 bp
Trp53 (P53)	For: 5'-CAGGGCTGAGACACAATC-3'<br>Rev: 5'-TGGGGTAGGGTGAGATTT-3'	201 bp
Pbx1	For: 5'- AAACTGCCACAGAATGAAGC-3'<br>Rev: 5'- CTTCTCCAGCTCTGTGTGGT-3'	298 bp
Pde4a	For: 5'- CACAGCCTCTGTGGAGAAGT -3'<br>Rev: 5'- GGAGAGTTTCCTCAGTGCAA -3'	305 bp
Pde4b	For: 5'- CCAGCTAGAGACCATCCAGA -3'<br>Rev: 5'- GAAGATGTTAAGGCCCCATT -3'	367 bp
Pecam1	For: 5'- TTGGCACAACAAACAAGCTA-3'<br>Rev: 5'- GAAATCTTCTCGCTGTTGGA-3'	292 bp
Sell	For: 5'-CGCTCATTCATCCCATTAAC-3'<br>Rev: 5'-GCAAGGAGTCTGAGTTTCCA-3'	224 bp
Selp	For: 5'- TCTTGGGAATTCCACCTACA-3'<br>Rev: 5'- GGGCAGGAAGTGATGTTATG-3'	339 bp
Sfp1 (Pu.1)	For: 5'-CGGATGACTTGGTTACTTACG-3'<br>Rev: 5'-GTAGGAAACCTGGTGACTGAG-3'	292 bp
Slamf1	For: 5'-TCCATGCCTCAGTTTCTCTC-3'<br>Rev: 5'-GGCAACTTTACAGCAGCATT-3'	264 bp
Vcam1	For: 5'-CTGTACATCCCTCCACAAGG-3'<br>Rev: 5'-ACACGTCAGAACAACCGAAT-3'	321 bp
