# Supplementary figures and images for: Hematopoietic Stem and Progenitor Cell Maintenance and Multiple Lineage Differentiation Is an Integral Function of NFATc1
Source: Cells. 2022 Jun 23;11(13):2012. doi: 10.3390/cells11132012 (PMC9265824; doi:10.3390/cells11132012)

# Supplementary Figure S1

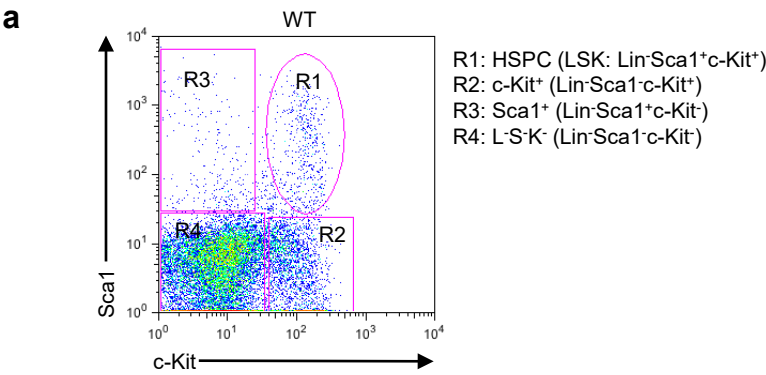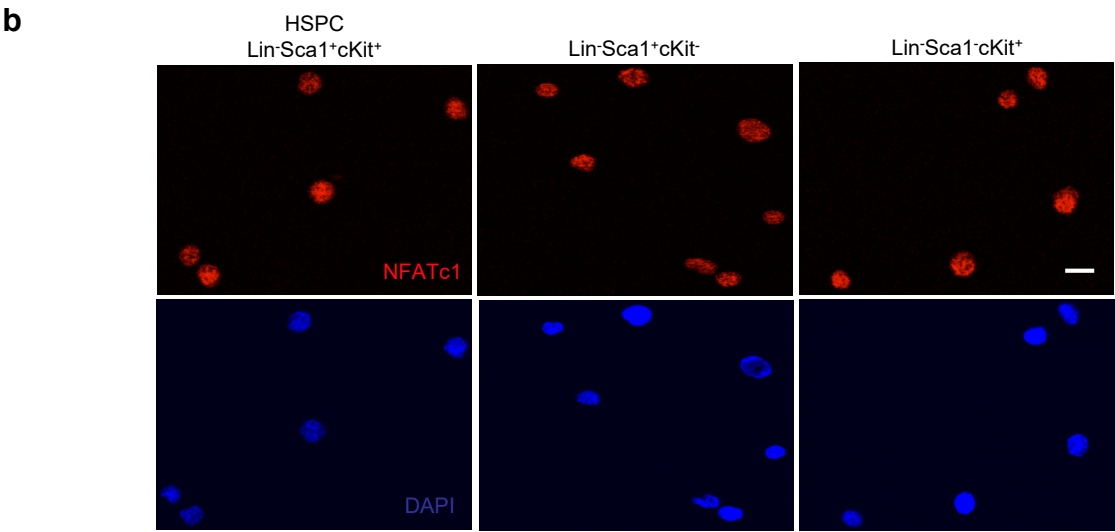

Supplement: Supplementary file 1 [file cells-11-02012-s001.zip › Patra_Supplementary Figure S1.pdf]

Supplementary Figure S2

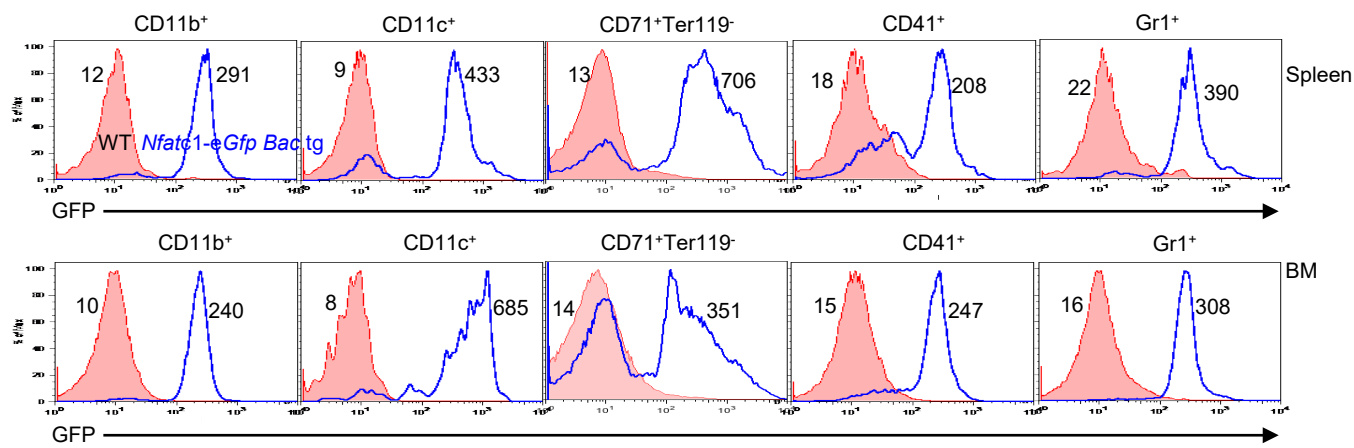

Supplement: Supplementary file 1 [file cells-11-02012-s001.zip › Patra_Supplementary Figure S2.pdf]

Supplementary Figure S3

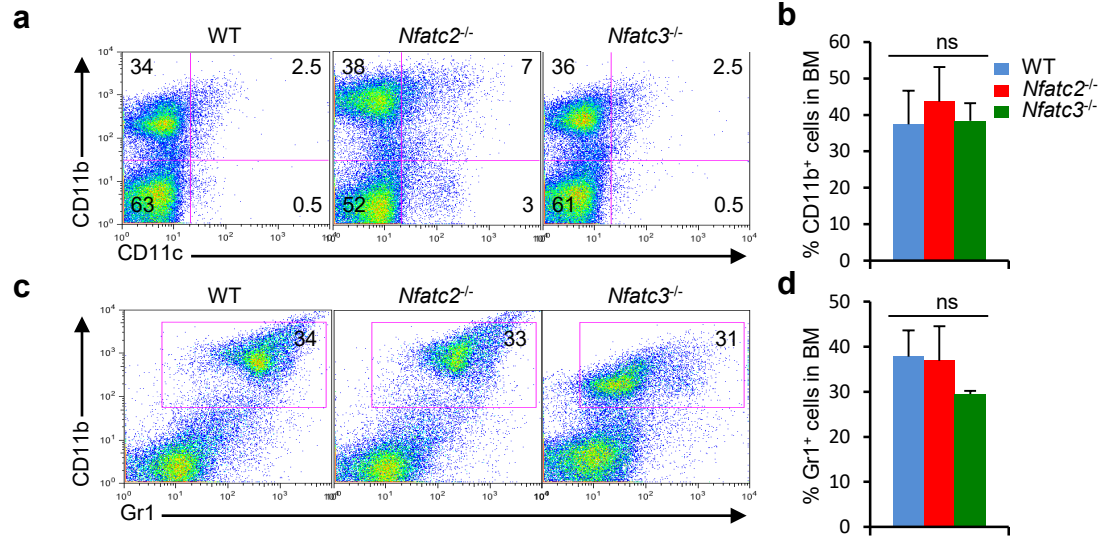

Supplement: Supplementary file 1 [file cells-11-02012-s001.zip › Patra_Supplementary Figure S3.pdf]

# Supplementary Figure S4

a

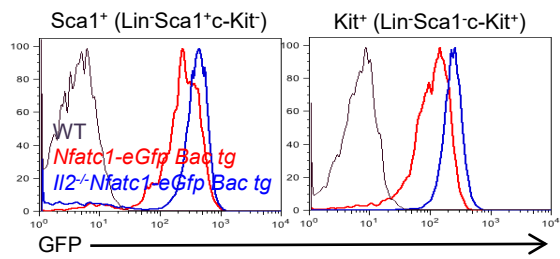

b

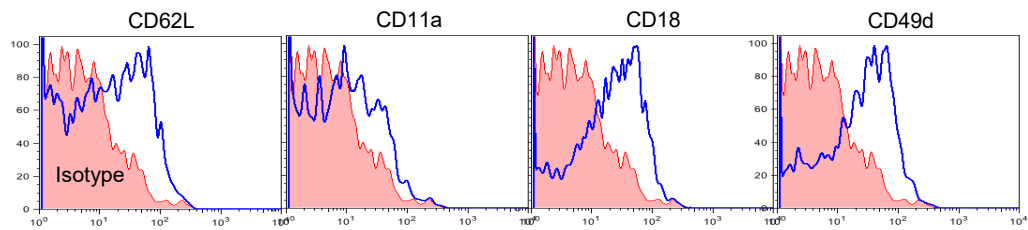

Supplement: Supplementary file 1 [file cells-11-02012-s001.zip › Patra_Supplementary Figure S4.pdf]
